# Review of The Physics and Technology of Radiation Therapy, 3^rd^ ed., by McDermott and Orton

**DOI:** 10.1002/acm2.70769

**Published:** 2026-08-25

**Authors:** Megan A. Hyun

**Affiliations:** ^1^ Department of Medical Physics Memorial Sloan Kettering Cancer Center New York New York USA

## 1 OVERVIEW

The 3^rd^ edition of The Physics and Technology of Radiation Therapy (2025) aims to introduce fundamental radiotherapy physics principles, together with their applications and related technologies, to an audience primarily consisting of radiation oncology residents. While this is the primary intended audience, the authors have designed the text to aid in teaching these same concepts to radiation therapist students, noting that the learning level should be appropriate for “the reader who has taken one year of college physics, perhaps years ago (and may not have been terribly thrilled about it).” As seems fitting, the two updates to the original textbook have each come 2–3 years following updates to the American Society for Radiation Oncology (ASTRO) Core Physics Curriculum for Radiation Oncology.^1^ In this review, I will discuss the merits of the text itself as well as its adherence to the 2023 ASTRO curriculum.

## 2 CHANGES IN THE 3RD EDITION

The textbook underwent only minor revision in its first half, which focuses on basic radiation physics principles. Some important content was added in this edition, including low‐dose‐rate (LDR) prostate seed implants (high‐dose‐rate prostate brachytherapy is not discussed), magnetic resonance (MR) linacs, total skin electron therapy, carbon ion therapy, convolution‐superposition dose calculation, and out‐of‐field or “peripheral” dose. Perhaps the most obvious change to readers familiar with the 2^nd^ edition is the removal of the appendix cross‐referencing standardized exam topics (e.g., American Registry of Radiologic Technologists exams, Medical Dosimetrist Certification Board exams) with sections of the text. While the authors do not mention this change in the preface, they note that the summaries at the end of each chapter have been expanded. The implication seems to be that readers ought to use their own judgement as to which chapters will help with specific exam topics, and that the chapter summaries are more appropriate study tools than the removed appendix.

## 3 CONTENT AND ORGANIZATION

This edition, like its predecessor, consists of 22 chapters. One of the most attractive features of the text for teaching and learning is its gradual progression in complexity.

The content progresses from reviews of basic math and physics (including helpful tips on using scientific calculators) through radiation production, interactions, and important related technologies. While the authors remark on the impossibility of covering every single topic recommended in the 2023 ASTRO Core without substantially lengthening the book, they cover the Core topics quite well. Notable exceptions include:

1. Electron beam characteristics and dosimetry
2. Advanced treatment planning and special procedures

While the text does include material on electron beam characteristics, it omits electron beam calibration due to the potential confusion of, for example, *k*_ecal_ and *k*_R50_, focusing instead on photon calibration using the original (prior to the 2014 addendum) American Association of Physicists in Medicine (AAPM) Task Group 51 (TG51) protocol.^2^, ^3^ Similarly, some treatment planning concepts are covered (e.g., dose distributions, beam modifiers, field matching, optimization), but the authors acknowledged in the preface to the 1^st^ edition that teaching treatment planning is outside the scope of the text.

There are three appendices in the 3^rd^ edition, which include answers to selected problems, dosimetric data (e.g., percent‐depth‐dose and tissue‐maximum‐ratio tables), and a dataset from a fictional linac called Mevalac. These resources help make the text come alive for the learner, making the content more interactive and instructional.

## 4 SIGNIFICANCE AND RELEVANCE

As an educator who has taught both radiation therapist students and radiation oncology residents, I appreciate the intentional design elements to make the text approachable and even, dare I say, fun. These elements include rules of thumb that “pop” on the page, clinical examples (even in basic physics chapters that might otherwise feel like a slog to some students), plenty of practice problems, and well‐organized chapter summaries that lay out the most important concepts of each chapter for effective studying. This is especially true in the 3^rd^ edition, where these summaries have been newly expanded.

Because of the close adherence to most of the Core topics, this textbook is an excellent resource for teaching physics to radiation oncology residents, especially if supplemented with additional resources to fill in the gaps. Even with the removal of the appendix connecting chapters to American Registry of Radiologic Technologists (ARRT) and Medical Dosimetrist Certification Board (MDCB) exam topics, it will also aid in teaching physics to radiation therapist and dosimetrist students. The application of the text to each of these learning groups may require some additional intentionality on the part of the educator. Medical physics trainees may find this textbook to be a helpful reference or study tool but should ultimately seek more in‐depth treatments of the topics during their training.

## 5 SUMMARY

The 3^rd^ edition of The Physics and Technology of Radiation Therapy by McDermott and Orton carries on the legacy of the previous editions in providing a clear and well‐organized introduction to radiation therapy physics appropriate for multiple learning groups. This edition adds some important technological updates without sacrificing a strong focus on the fundamentals, and is, as far as textbooks go, an enjoyable read.

## 6 ABOUT THE AUTHOR

Megan Hyun, PhD, DABR is an Associate Attending Physicist and Therapeutic Medical Physics Residency Program Director at Memorial Sloan Kettering Cancer Center in New York, NY.
